# Treatment of internuclear ophthalmoparesis in multiple sclerosis with fampridine: A randomized double‐blind, placebo‐controlled cross‐over trial

**DOI:** 10.1111/cns.13096

**Published:** 2019-02-12

**Authors:** Kawita M. S. Kanhai, Jenny A. Nij Bijvank, Yorick L. Wagenaar, Erica S. Klaassen, KyoungSoo Lim, Sandrin C. Bergheanu, Axel Petzold, Ajay Verma, Jacob Hesterman, Mike P. Wattjes, Bernard M. J. Uitdehaag, Laurentius J. van Rijn, Geert Jan Groeneveld

**Affiliations:** ^1^ Centre for Human Drug Research Leiden the Netherlands; ^2^ Department of Ophthalmology Neuro‐ophthalmology Expertise Center Amsterdam UMC Amsterdam the Netherlands; ^3^ Department of Neurology MS Center and Neuro‐ophthalmology Expertise Center Amsterdam UMC Amsterdam the Netherlands; ^4^ KCRN Research Germantown Maryland; ^5^ The National Hospital for Neurology and Neurosurgery Queen Square and Moorfields Eye Hospital London UK; ^6^ Experimental Medicine Biogen Cambridge Massachusetts; ^7^ inviCRO Boston Massachusetts; ^8^ Department of Radiology and Nuclear Medicine Amsterdam University Medical Center Amsterdam the Netherlands; ^9^ Department of Ophthalmology Onze Lieve Vrouwe Gasthuis Amsterdam the Netherlands

**Keywords:** fampridine, internuclear ophthalmoplegia, multiple sclerosis, video‐oculography

## Abstract

**Aim:**

To examine whether the velocity of saccadic eye movements in internuclear ophthalmoparesis (INO) improves with fampridine treatment in patients with multiple sclerosis (MS).

**Methods:**

Randomized, double‐blind, placebo‐controlled, cross‐over trial with fampridine in patients with MS and INO. Horizontal saccades were recorded at baseline and at multiple time points post‐dose. Main outcome measures were the change of peak velocity versional dysconjugacy index (PV‐VDI) and first‐pass amplitude VDI (FPA‐VDI). Both parameters were compared between fampridine and placebo using a mixed model analysis of variance taking patients as their own control. Pharmacokinetics was determined by serial blood sampling.

**Results:**

Thirteen patients had a bilateral and 10 had a unilateral INO. One patient had an INO of abduction (posterior INO of Lutz) and was excluded. Fampridine significantly reduced both PV‐VDI (−17.4%, 95% CI: −22.4%, −12.1%; *P* < 0.0001) and FPA‐VDI (−12.5%, 95% CI: −18.9%, −5.5%; *P *< 0.01). Pharmacokinetics demonstrated that testing coincided with the average *t*_max_ at 2.08 hours (SD 45 minutes). The main adverse event reported after administration of fampridine was dizziness (61%).

**Conclusion:**

Fampridine improves saccadic eye movements due to INO in MS. Treatment response to fampridine may gauge patient selection for inclusion to remyelination strategies in MS using saccadic eye movements as primary outcome measure.

## 1 INTRODUCTION

Successful remyelination remains the Holy Grail for the treatment of multiple sclerosis (MS), a predominantly demyelinating disease. In a proof‐of‐principle study, it has been shown that Clemastine fumarate has the potential to induce remyelination of the optic nerve.^1^ Following treatment with clemastine, conduction velocities in patients with long‐standing optic neuritis improved compared to placebo. Optic neuritis has been the dominant model for testing remyelination strategies in MS.^2^ In optic neuritis, there is consistent evidence for substantial and irreversible axonal loss.^3^ This had been recognized to limit the chance for successful remyelination, and severe retinal nerve fiber loss (>70 μm) was an exclusion criterion in the ReBUILD trial.^1^ Other limitations of optic neuritis for testing remyelination encompass that recovery of high contrast visual acuities is typically excellent even without treatment, the need for combined assessment of electroretinogram (ERG), and visual evoked potential (VEP) in order to be compliant with international standards, which not all patients tolerate.^4^ Finally, results from an optic neuritis treatment trial may require to be cross‐validated by another model for remyelination in MS which has different, validated outcome measures.

Demyelination in MS does affect a functionally relevant single axon pathway, the medial longitudinal fasciculus (MLF).^5^ A MS lesion in the MLF causes an internuclear ophthalmoparesis which can be aggravated though Uhthoff's phenomenon indicating functioning axons with alterable conduction velocities.^6^, ^7^ Rapid (saccadic) horizontal adducting eye movements are impaired in an INO, and patients report transient diplopia, visual confusion, the illusion of environmental movements during a saccade, vertigo, and a transient blur, for example, while reading.^8^, ^9^ The prevalence of clinically evident INO in MS ranges from 24% to 55% and are bilateral in most cases.^8^, ^10^, ^11^, ^12^, ^13^ Anatomically, the MLF is located close to the floor of the fourth ventricle and readily discernible on MRI. The MLF represents an efferent pathway which signals from the sixth nucleus to the third nucleus in order to enable coordinated horizontal eye movements.^9^ These saccadic eye movements can be easily quantified by infrared oculography (eye tracking). We have developed and validated a novel protocol for assessment of horizontal saccadic eye movements suitable for a multicenter setting with excellent parameter reproducibility.^14^ For the most commonly used parameters for testing an INO, the intra‐class correlation coefficients (ICC) were >0.9 for the peak velocity versional dysconjugacy index (PV‐VDI) and the first‐pass amplitude VDI (FPA‐VDI).^14^

In this study, we tested whether a drug known to accelerate nerve conduction, fampridine (dalfampridine), has an effect on saccadic eye movements in patients with MS who suffer from an INO. Previously fampridine, a voltage‐gated potassium channel blocker was shown to improve walking speed in patients with MS.^15^ A year later, fampridine was approved by the FDA for treating impaired walking in MS patients. Since, the anecdotal evidence has been published showing three cases of oral Fampridine (10 mg) improving horizontal saccades in INO.^16^ This double‐blind, placebo‐controlled cross‐over study demonstrates that fampridine significantly improved our two carefully validated^14^ primary outcome measures, the PV‐VDI, and FPA‐VDI.

## 2 MATERIALS AND METHODS

This single‐center, double‐blind, randomized placebo‐controlled cross‐over study was conducted by the Centre for Human Drug Research (Leiden, the Netherlands) between April 2015 and August 2016. This study was approved by the Medical Ethics Committee of the BEBO Foundation (Assen, the Netherlands) and was conducted in accordance with the Dutch Act on Medical Research Involving Human Subjects (WMO) and in compliance with Good Clinical Practices (ICH‐GCP) and the Declaration of Helsinki. This study was registered in the European Union Clinical Trials Register (protocol number 2015‐000182‐31) and in the Dutch Clinical Trial Registry (www.toetsingonline.nl; dossier number NL52195.056.15). All patients were recruited and assessed at the Amsterdam UMC (Amsterdam, the Netherlands). All patients had to provide written consent prior to being considered for inclusion.

Patients were approached by their neurologists, through existing research projects and advertisements on the hospital website. Of 28 eligible patients, two decided not to participate for personal reasons.

Twenty‐four patients with MS who had a clinically evident or suspected INO participated in an initial screening visit and two further trial occasions. Each subject was randomly assigned to receive either fampridine (20 mg) on occasion 1 and placebo on occasion 2 or placebo on occasion 1 and fampridine on occasion 2. The interval between the occasions was at least 1 week, which is more than 5 half‐lives of fampridine, thereby guaranteeing an adequate washout.

All patients had a diagnosis of clinically definite MS based on the 2010 revision of the McDonald criteria.^17^ The disease duration had to be longer than 1 year and the time from last relapse at least 30 days. All patients were screened using a prosaccadic task. The presence of INO (unilateral or bilateral) was determined by visual examination of the graphs depicting horizontal gaze position by two experienced observers (JANB, KMSK). The inter‐rater agreement was 95.8% (23/24). The one patient in whom there was a disagreement had an INO of abduction and was excluded from this trial. This case has been published separately.^18^ All patients were otherwise healthy, including normal ECG, hematology, and blood chemistry; all patients were also seronegative for HIV, hepatitis B, and hepatitis C, had no history of seizures, normal creatinine clearance, no contraindications for MRI, and no concomitant use either an inhibitor or substrate of organic cation transporter 2.^19^

The randomization was created by an independent statistician at the Centre for Human Drug Research using SAS version 9.4. Only independent staff members at the trial pharmacy of the Amsterdam University Medical Center were unblinded; all patients, investigators, and study coordinators were blinded with respect to treatment assignment.

The **s**tudy medication was fampridine (Biogen). The most commonly prescribed daily dose of fampridine is 20 mg. To ensure optimal plasma levels through the day and reduce the risk of adverse events, fampridine is usually taken in two daily doses of 10 mg each. Because we were interested in studying the acute effects of a single dose of fampridine on INO, we administered a single 20 mg dose of fampridine in order to increase the likelihood of demonstrating a pharmacodynamic effect by achieving pharmacologically active yet safe plasma concentrations. Based on published literature, the risk of adverse events following a single 20 mg dose of fampridine is acceptable.^20^ Subjects were instructed not to eat within one hour before dosing and one hour after dosing.

Table S1 summarizes the schedule and timing of all assessments. The safety evaluation included the recording of all adverse events and measurements of blood pressure and heart rate.

The pharmacokinetics of fampridine was assessed on serial blood plasma samples. Blood was collected prior to dosing and 1.5, 2, 2.5, 3, 3.75, 4.25, and 5.25 hours after dosing. Blood was drawn into 4‐mL EDTA tubes and centrifuged at 2000 *g* for 10 minutes at 2‐8°C; the plasma fractions were then transferred to 2‐mL tubes and were stored at −20°C within 30 minutes of sampling. Plasma fampridine concentration was measured using ultra‐performance liquid chromatography‐tandem mass spectrometry (UPLC‐MS/MS). Descriptive pharmacokinetics included the average time to reach maximum concentration (*t*_max_), the average maximum concentration (*C*_max_), and the average area under the curve from dosing until the final measurement (AUC_0‐last_).

Eye movements were recorded by video‐oculography using the EyeLink 1000 Plus eye‐tracking system (SR Research, Ottawa, Canada). The setup of the full protocol, including also fixation stability, antisaccades, and double step saccades in addition to a range of prosaccadic tasks, has been described in detail.^14^ For this study, an abbreviated version was used. In brief, for testing, the head was stabilized by a chin and forehead rest. The system uses the pupil and corneal reflection to determine the eye position at different eccentricities at 1000 Hz sampling frequency. A calibration procedure was performed prior to each assessment.“The prosaccade task consisted of 10 trials of 8 horizontal prosaccades.” prosaccade task consisted of 10 trials of 8 horizontal prosaccades. Centrifugal saccades were analyzed from the center of the screen to an eccentric location either 6.25 or 12.5 degrees of visual angle to the left or right. The task lasted approximately 15 minutes and was performed prior to dosing and 1.5, 2.5, 3.75, and 5.5 hours after dosing.

The data were analyzed off‐line using custom‐made software written in MATLAB (MathWorks, Natick, MA). For each correct centrifugal saccade, peak velocity (PV) and the first‐pass amplitude (FPA) were determined. The FPA was defined as the amplitude of the eye at the time point at which the abducting eye first reached the target position.^21^, ^22^ The versional dysconjugacy index (VDI) was then calculated for both PV and FPA by dividing the abducting eye's value by the adducting eye's value 14, 23 (see Supplementary S5, S6, and S7 for additional explanation). The unity of the VDI is normal.

Brain imaging was performed by magnetic resonance imaging (MRI). We used a 3 Tesla machine (Discovery MR750 3.0T whole‐body MR system, GE Healthcare). A multisequence MRI protocol was performed, including axial T1‐weighted spin echo, axial proton density (PD), T2‐weighted fast spin echo, 3D fluid‐attenuated inversion recovery (FLAIR), coronal T2 short tau inversion recovery (STIR), 3D T1‐weighted fast‐spoiled gradient echo (FSPGR), and axial diffusion tensor imaging (DTI) sequences. Data analysis included image preprocessing and segmentation steps to generate MLF and MS lesion masks, which comprised six unique regions of interest, including the entire left/right MLF, the lesioned left/right MLF, and the nonlesioned left/right MLF. Volume, length, and DTI‐based scalars (median/axial/radial diffusivity and fractional anisotropy) were estimated for each of the six regions of interest. The MLF lesion load was determined. The scans were reviewed by two researchers. An example MRI image is shown in Figure S8.

All statistical analyses were performed in SAS version 9.4 (SAS Institute Inc., Cary, NC, USA). To determine whether significant treatment effects could be detected using the saccadic protocol, the PV‐VDI and FPA‐VDI were analyzed using a mixed model analysis of covariance (ANCOVA). Treatment, time, period, and treatment by time were used as fixed factors; subject, subject by treatment, and subject by time were used as random factors, and the average baseline measurement (pre‐fampridine) per period was used as a covariate. Due to their log‐normal distribution, the PV‐VDI and FPA‐VDI parameters were log‐transformed prior to analysis. Any change relative to baseline value was analyzed using the same model for graphical purposes. We used the Spearman rank correlation test to measure the correlation between baseline characteristics and treatment conditions. For the eye‐tracking tests, individual INOs were treated as individual subjects in both the ANCOVA and Spearman rank correlation test. The null hypothesis was rejected if *P* < 0.05. The alternative hypothesis was 2‐sided.

## 3 RESULTS

**Figure 1:**
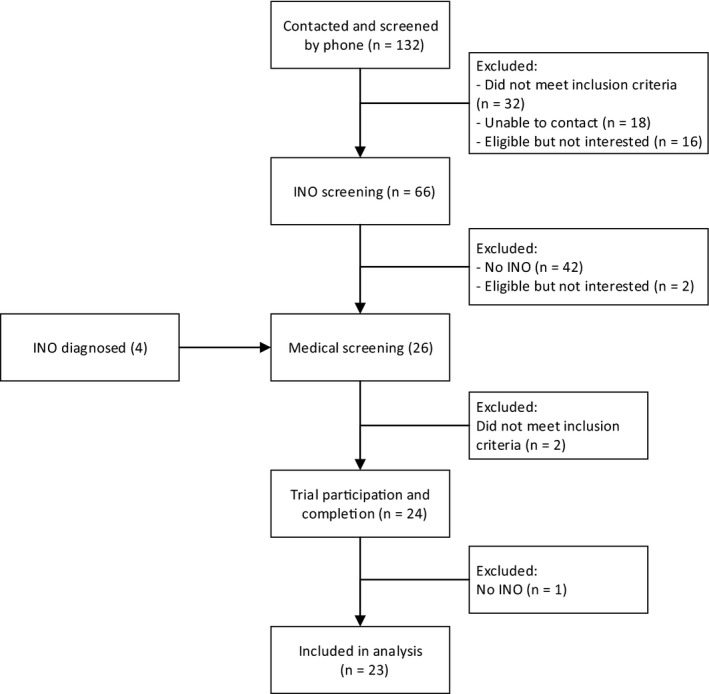
CONSORT flow diagram for the randomized controlled trial

A total of 24 subjects participated and completed the study between April 2015 and August 2016 (see Figure 1 for the CONSORT diagram). One patient was diagnosed with a rare posterior INO of Lutz and excluded from further analysis. We included 11 male and 12 female patients (Table 1). The INO was bilateral in 13 and unilateral in ten.

**Table 1:** Subject characteristics

Characteristic	Patients (n = 23)
Age, mean (min‐max), years	49 (31‐72)
No. (%) females	12 (52)
Disease duration, mean (min‐max) years	12.6 (1‐25)
Weight, mean (min‐max) kg	71.9 (45.1‐103)
Height, mean (min‐max) cm	176 (157‐188)
BMI, mean (min‐max) kg/m2	23.1 (17.2‐29.4)
No. (%) bilateral INO	13 (56)

The most commonly reported adverse event (AE) was dizziness 60.8% (14/23). This was followed by fatigue (3 fampridine, 5 placebo) and headache (2 fampridine, 2 placebo). Fampridine had no effect on blood pressure or heart rate. All reported AEs are listed in Table S9.

**Figure 2:**
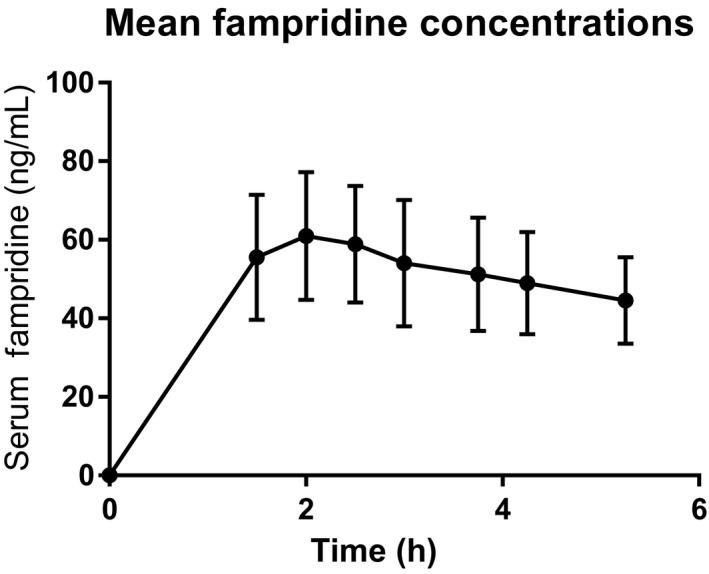
Mean fampridine serum concentrations with SD, measured in the subjects during the occasion

Descriptive pharmacokinetics revealed a mean (±SD) *t*_max_ of 128 ± 45 minutes), a mean *C*_max_ of 65 ± 15 ng/mL, and a mean AUC_0‐last_ of 220 ± 57 ng*hr/mL. The plasma fampridine concentration over time is shown in Figure 2.

**Figure 3:**
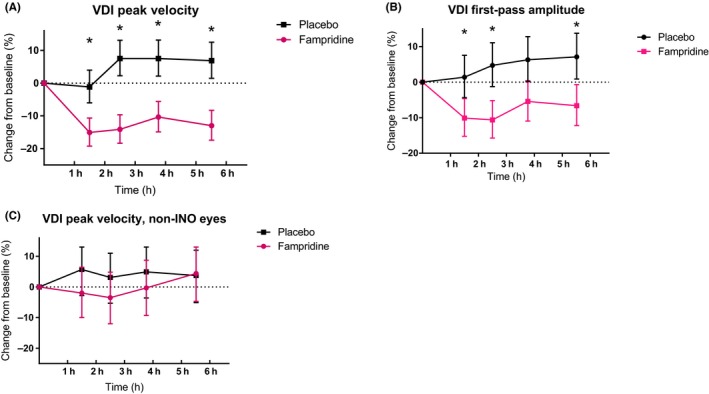
Pharmacodynamic results. (A) Shows the VDI peak velocity change from baseline least square means with upper and lower limit. (B) Shows the VDI first‐pass amplitude change from baseline least square means with upper and lower limit. (C) Shows the mean change from baseline least square means in VDI peak velocity for the non‐INO eyes with upper and lower limit. Significant differences are indicated by an asterisk (*)

Fampridine improved saccadic eye movements of the INO eyes in all 23 patients (Figure 3A). This change was observed from the first measurement after dosing (1.5 hours after dosing) until the last measurement (5.5 hours after dosing). The effect of fampridine on the PV‐VDI was significant if compared to placebo. The difference from baseline calculated to −17.4% (95% CI: −22.4%, −12.1%; *P* < 0.0001, Table 2). Similar results were obtained for FPA‐VDI (Figure 3B), with an estimated difference of −12.1% (95% CI: −17.6%, −6.2%; *P* < 0.001, Table 2).

**Table 2:** Analysis results table video‐oculography

Pharmacodynamic measurement	Estimate of the difference (%)	95% CI		*P*‐value
		Lower (%)	Upper (%)	
VDI peak velocity (%), eyes with INO	−17.4	−22.4	−12.1	<0.0001
VDI peak velocity (%), eyes without INO	−4.6	−10.3	1.5	0.105
VDI first‐pass amplitude (%), eyes with INO	−12.1	−17.6	−6.2	0.0003

There was no significant effect of fampridine on non‐INO eyes (n = 10). The difference from baseline calculated to −4.6% (95% CI: −10.3%, 1.5%; *P* = 0.105, Table 2 and Figure 3c).

The magnitude of improvement of saccadic eye movements in INO was directly correlated to the PV‐VDI and FPA‐VDI at baseline (ρ = −0.35, *P* = 0.0345; Figure S2). There was no such correlation with disease duration (*ρ* = 0.02, *P* = 0.909; Figure S3) or the MRI lesion load of the MLF (*ρ* = 0.08, *P* = 0.658; Figure S4).

## 4 CONCLUSION

To our knowledge, this clinical trial together with prior pharmacological, pathological, and clinical studies^7^, ^8^, ^9^, ^16^, ^23^, ^24^ provides evidence that fampridine significantly improves the PV‐VDI and FPA‐VDI with a single dose of 20 mg within 1.5 hours. The response is sustained for at least 5 hours which parallels the robust pharmacodynamic data over this period. The most common AE was dizziness in 60.8% of all patients.

The effect of fampridine appears to be selective for the demyelinated axons of the MLF, corroborated by MRI data, of INO eyes, and absent in non‐INO eyes. There appears also to exist a linear relationship between the degree of impairment of saccadic eye movements and the degree of improvement with treatment. These findings were independent to other covariates such as disease duration or MRI lesion load. The lack for a correlation of functional improvement with MRI metrics has been recognized before 25 and poses a challenge for clinical trials.^26^ In this context, present data provide a valuable, reproducible,^14^ primary outcome measure suitable for testing remyelination strategies in INO in MS.

Our pharmacological data also suggest that the treatment effect of fampridine on eye movements already becomes significant prior to reaching *t*_max_ after about 2 hours. Based on this and a prior trial on the effect of fampridine on walking speed,^15^ we suspect that a lower dose of 10 mg may be sufficient. A lower dose may also be better tolerated by patients, the majority of whom did suffer from drug‐related dizziness. The proportion of patients with dizziness in this study was higher than the 10%‐20% expected from standard dosing. The prevalence of other AEs, including headache and fatigue, was similar between the placebo and fampridine occasions.

The findings of present trial are consistent with Serra's detailed study of the effect of 10 mg dalfampridine in three cases of INO.^16^ Each, but one of the patients did show “significant decrease of saccadic abduction/adducting eye peak velocities for the worst INO” after treatment with fampridine.^16^ In other words, fampridine reduces on a patient level the pathological slowing of the adducting eye in an INO. Furthermore, the pharmacodynamic data exclude the possibility of a type II errors which could be caused by low/absent fampridine blood levels.

There is good preclinical data supportive of the observations reported in this trial. Waxman and others have clearly demonstrated that ion channels are redistributed along demyelinated axons during regeneration.^27^, ^28^ Of these, redistribution of potassium channels has the disadvantage of reducing the generation of axon potentials by accelerating membrane repolarization. Fampridine directly affects demyelinated nerves by blocking potassium channels and therefore promotes conduction in demyelinated axons.^29^

These experimental data also imply that there are other potassium channel drugs which may show similar effects. For example, the structure of fampridine (4‐aminopyridine) is similar to the structure of 3,4‐diaminopyridine. The reason for using fampridine for this trial was that it is FDA approved for treatment in MS. A limitation is that fampridine is not licensed for the treatment of an INO and we would be hesitant to recommend it as a routine treatment, least of all because of side effects.

Another limitation of this study is that we did not investigate the effect of fampridine on functional outcomes of vision in MS. One reason is that there is no validated questionnaire for the visual problems specifically caused by an INO. These symptoms can be difficult to be explained by patients. A further limitation is that we did not include a visual quality of life measure such as the National Eye Institute Visual Function Questionnaire (NEI‐VFQ‐25).

Taken together, the main advantage of this trial is that it provides evidence that a chronic INO is helpful in expanding the number of patients to include in trials testing remyelinating strategies in MS. Testing of saccadic eye movements are a validated, highly reproducible primary outcome measures,^5^, ^30^ and suitable for a multi‐center setting. We believe this will be important to cross‐validate findings on remyelination in optic neuritis which rely on a range of visual evoked visual potential test paradigms and optical coherence tomography as an outcome measures.^1^, ^31^ The prevalence of INO in MS appears to be high enough, 24%‐55% to ensure that there will sufficient patients suitable for trials. 14

## CONFLICT OF INTEREST

KK, JNB, YW, EK, KSL, SB, LJRvR, and GJG declare no conflict of interest. JH is an employee at InviCRO, which receives funding from Biogen for MRI analyses and was contracted by CHDR to perform the analyses in this study. AP reports personal fees from Novartis, grants from Novartis, outside the submitted work; and AP is part of the steering committee of the OCTiMS study which is sponsored by Novartis. He has not received honoraria for this activity. AP is supported by the National Institute for Health Research (NIHR) Biomedical Research Centre based at Moorfields Eye Hospital National Health Service (NHS) Foundation Trust and University College London Institute of Ophthalmology. AV was an employee at Biogen during the study. MPW received both speaking and consultancy fees from Biogen, Bracco, Genzyme, IXICO, Novartis, and Roche. BU received compensation for consulting from Biogen, Genzyme, Merck Serono, Novartis, Roche, and Teva Pharmaceuticals.
