# Supplementary material for: Treatment of internuclear ophthalmoparesis in multiple sclerosis with fampridine: A randomized double‐blind, placebo‐controlled cross‐over trial
Source: CNS Neurosci Ther. 2019 Feb 12;25(6):697–703. doi: 10.1111/cns.13096 (PMC6515699; doi:10.1111/cns.13096)
Supplement: Supplementary file 1 [file CNS-25-697-s001.docx]

**Supplemental material**

S1. Visit and Assessment Schedule

|  | SCR | Treatment days 1, 8 | | | | | | | | |  |
| --- | --- | --- | --- | --- | --- | --- | --- | --- | --- | --- | --- |
| Time point  Assessment | Up to  -21 d | -1  h | 0  h | 1.5  h | 2  h | 2.5  h | 3  h | 3.75  h | 4.25  h | 5.5h | |
| Informed consent | X |  |  |  |  |  |  |  |  |  | |
| Demography | X |  |  |  |  |  |  |  |  |  | |
| Inclusion and exclusion criteria | X |  |  |  |  |  |  |  |  |  | |
| Medical history | X |  |  |  |  |  |  |  |  |  | |
| Physical examination | X |  |  |  |  |  |  |  |  |  | |
| Concomitant medication | X | X |  |  |  |  |  |  |  |  | |
| Meals |  |  |  |  | X |  |  |  |  |  | |
| Virology | X |  |  |  |  |  |  |  |  |  | |
| BsHaem, BsChem, Urinalysis | X |  |  |  |  |  |  |  |  |  | |
| UrDrug, BrAlc | X |  |  |  |  |  |  |  |  |  | |
| ECG | X |  |  |  |  |  |  |  |  |  | |
| Vital Signs (BP, HR) | X | X |  | X |  | X |  | X |  | X | |
| Drug (-placebo) administration |  |  | X |  |  |  |  |  |  |  | |
| 3T MRI | X |  |  |  |  |  |  |  |  |  | |
| PK sample |  | X |  | X | X | X | X | X | X | X | |
| Eye Tracking Test | X | X^[[1]](#footnote-1)^ |  | X |  | X |  | X |  | X | |
| NeuroCart test battery | X^[[2]](#footnote-2)^ | X^[[3]](#footnote-3)^ |  |  |  |  | X |  | X |  | |
| - Pharmaco-EEG |  | X |  |  |  |  | X |  | X |  | |
| - Simple reaction time task | X | X |  |  |  |  | X |  | X |  | |
| - Adaptive tracking | X | X |  |  |  |  | X |  | X |  | |
| - Rapid visual information processing | X | X |  |  |  |  | X |  | X |  | |
| - Body sway | X | X |  |  |  |  | X |  | X |  | |
| - Symbol digit substitution test | X | X |  |  |  |  | X |  | X |  | |
| National Eye Institute visual functioning questionnaire (VFQ-25) |  | X |  |  |  |  |  |  |  |  | |
| Discharge |  |  |  |  |  |  |  |  |  | X | |
| (S)AE |  | <----- continuous -----> | | | | | | | | |  |

SCR = Screening, BsHaem = Blood Sample Haematology, BsChem = Blood Sample Chemistry, UrDrug = Urine Drug Screen, ECG = Electrocardiogram,
BP = Blood Pressure, HR = Heart Rate, MRI = Magnetic Resonance Imaging, MLF = Medial Longitudinal Fasciculus, AE = Adverse Event,
SAE=Serious AE


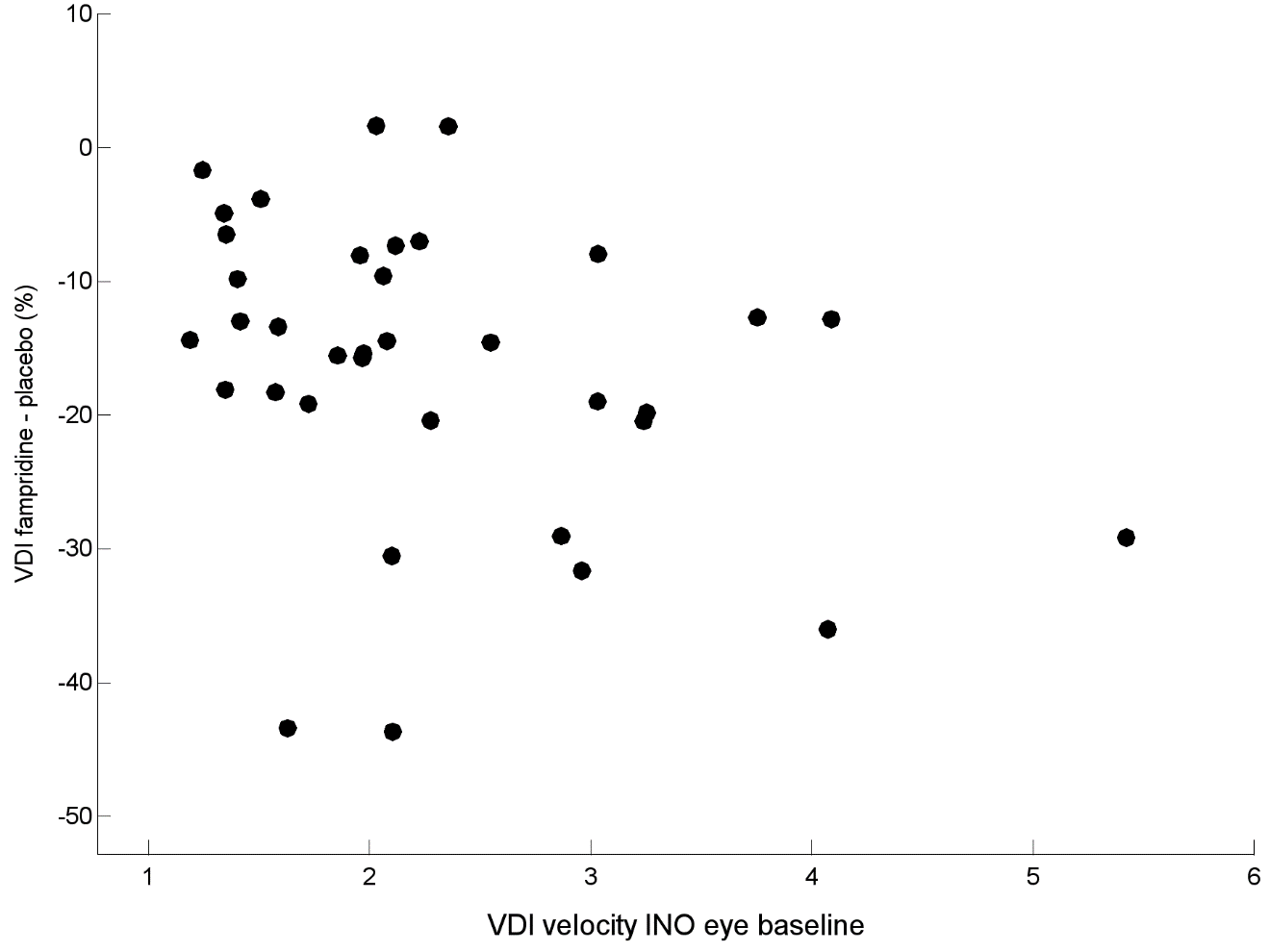


S2. Scatter plot showing the peak velocity VDI at baseline versus peak velocity VDI difference between fampridine and placebo (average difference over all time points). VDI was calculated for every eye with INO (n=36; some patients have a bilateral INO).


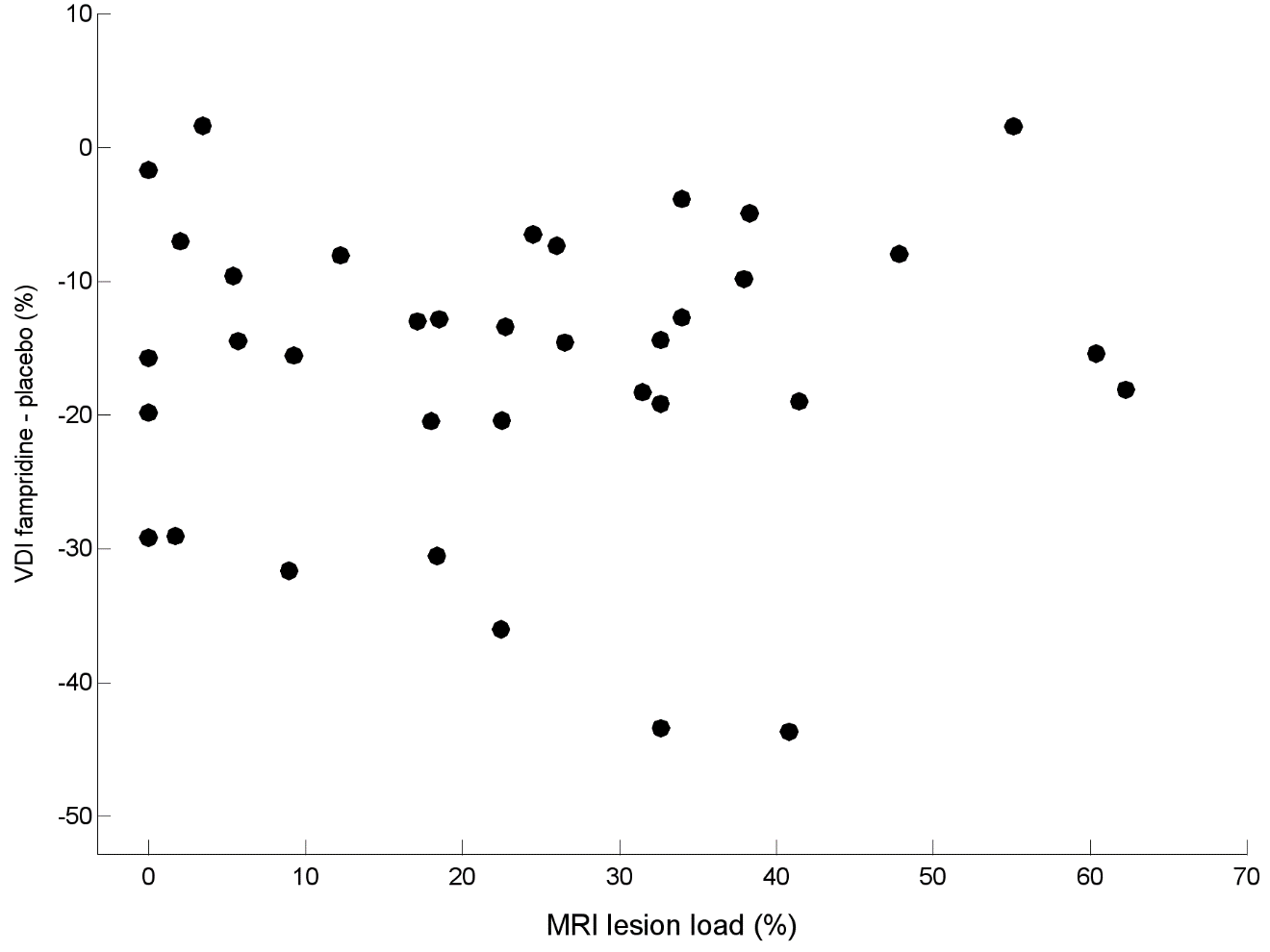


S3. Scatter plot showing the MRI lesion load (% of MLF) versus peak velocity VDI difference between fampridine and placebo (average difference over all time points). VDI was calculated for every eye with INO (n=36; some patients have a bilateral INO).


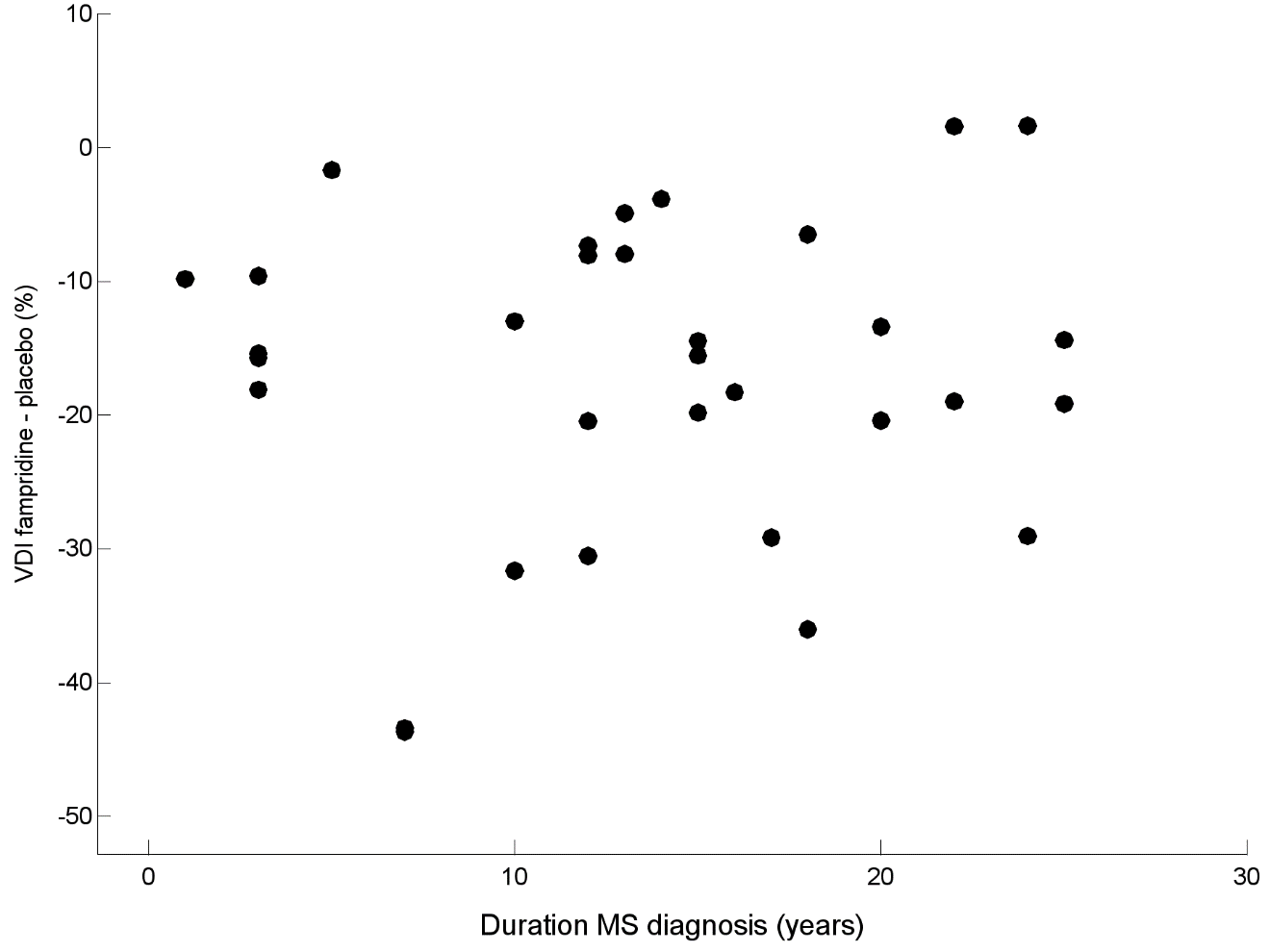


S4. Scatter plot showing the duration of disease (number of years since diagnosis) versus peak velocity VDI difference between fampridine and placebo (average difference over all time points). VDI was calculated for every eye with INO (n=36; some patients have a bilateral INO).


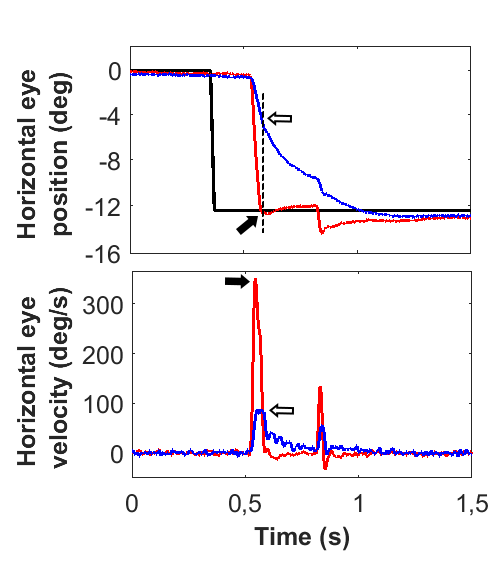


S5. Horizontal eye position (upper graph, degrees of visual angle) and eye velocity (lower graph, degrees of visual angle per second) of a leftward saccade of a MS patient with INO, showing the FPA and peak velocity. In the upper graph, the solid black line represents the target position. In both graphs, the blue line represents the right eye and the red line represents the left eye.
a. The dashed line indicates the time point where the abducting eye first reaches the target position. The black arrow points out the eye position of the abducting eye at this time point, the black-lined arrow the eye position of the adducting eye. The FPA is the amplitude of an eye at this time point.
b. The black arrow points out the peak velocity of the abducting eye during the centripetal saccade, the black-lined arrow the peak velocity of the adducting eye.

S6. Horizontal eye position (degrees of visual angle) of two centripetal saccades in a healthy volunteer (a.), a MS patients with unilateral INO (b.) and a MS patient with bilateral INO (c.). The solid black line represents the target position, the blue line the right eye and the red line the left eye. For the horizontal eye position, value zero corresponds to the center of the screen, a positive value to a position at the right side of the center and a negative value to a position at the left side of the center.


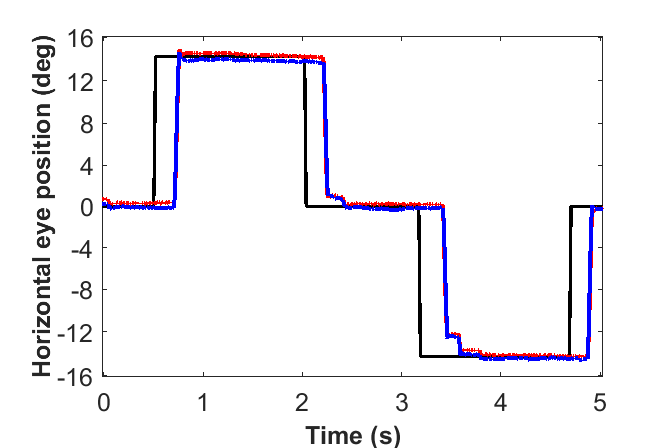

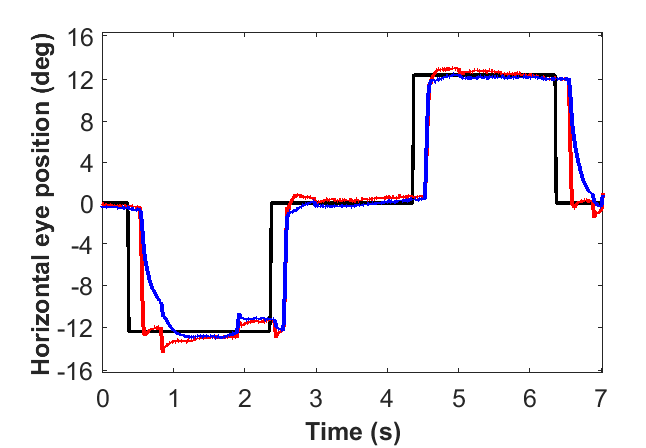

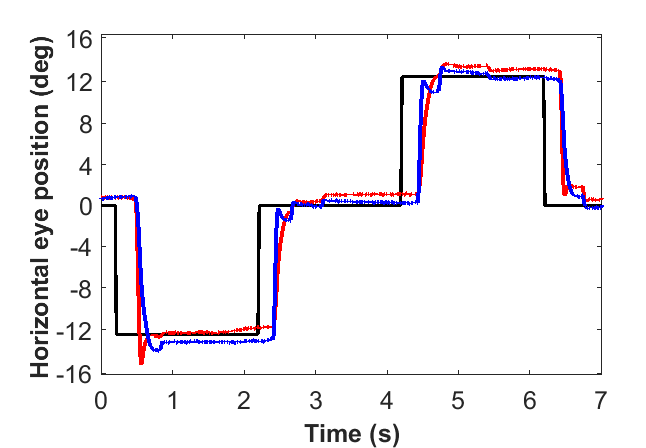


b.

c.

a.

a.

b.


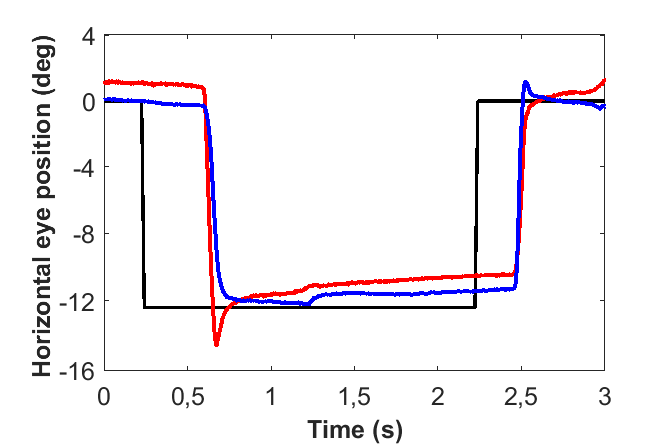

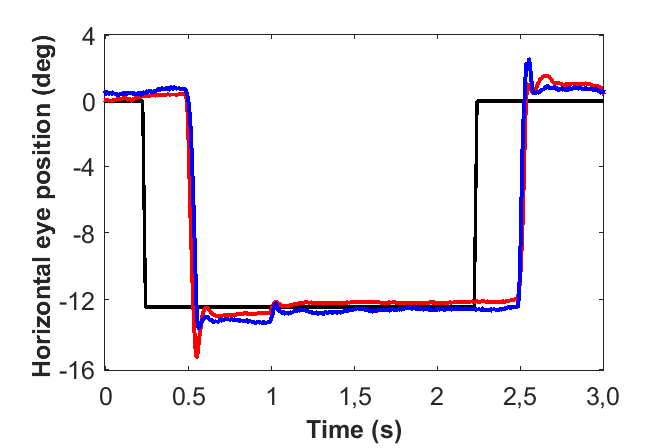


S7. Horizontal eye position (degrees of visual angle) of a leftward centripetal saccades in a MS patients with a bilateral INO (subject 2), pre-dose (a.) and 1.5 hours post-dose (b.). The solid black line represents the target position, the blue line the right eye and the red line the left eye.

S8. Axial T2-weighted image at the level of the pons showing a small periventicular lesion affecting the right MLF traject (arrow)


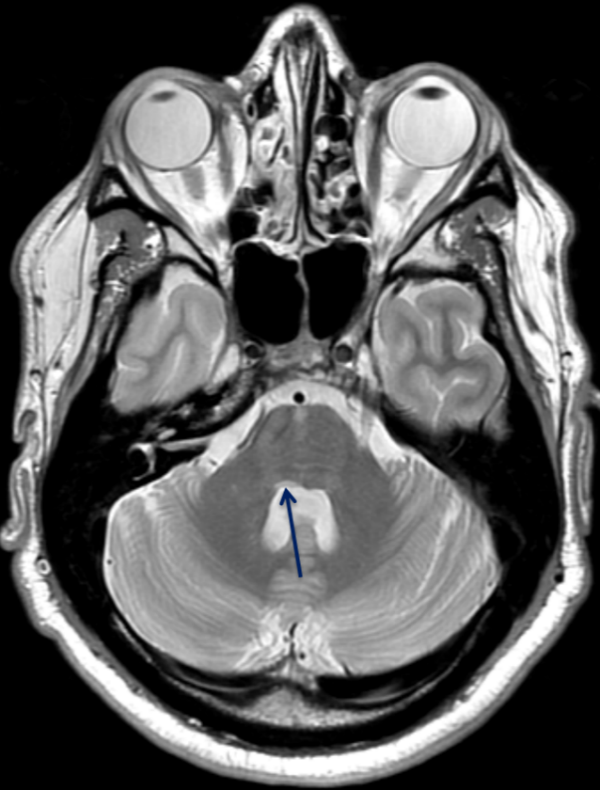


S9. All reported adverse events (including those not related to the treatment).

| **Adverse event** | **Fampridine**  (number of subject with the AE  (% of total number of subjects)) | **Placebo**  (number of subject with the AE  (% of total number of subjects)) |
| --- | --- | --- |
| Dizziness | 14 (61%) |  |
| Fatigue | 3 (13%) | 5 (22%) |
| (Resting) tremor | 2 (9%) |  |
| Headache | 2 (9%) | 2 (9%) |
| Hyperhidrosis | 2 (9%) |  |
| Asthenia | 1 (4%) |  |
| Back pain | 1 (4%) |  |
| Chest discomfort | 1 (4%) |  |
| Diarrhoea | 1 (4%) |  |
| Nausea | 1 (4%) |  |
| Paraesthesia | 1 (4%) |  |
| Restless legs syndrome | 1 (4%) |  |
| Somnolence | 1 (4%) | 1 (4%) |
| Visual impairment | 1 (4%) |  |
| Nasopharyngitis |  | 1 (4%) |

S10. Fampridine concentrations (ng/mL) over time for a single 20mg dose (blue) or a twice-daily 10mg dose at steady-state (magenta), predicted using the pharmacokinetic parameters from Weir et al (Curr Med Res Opin. 2013).


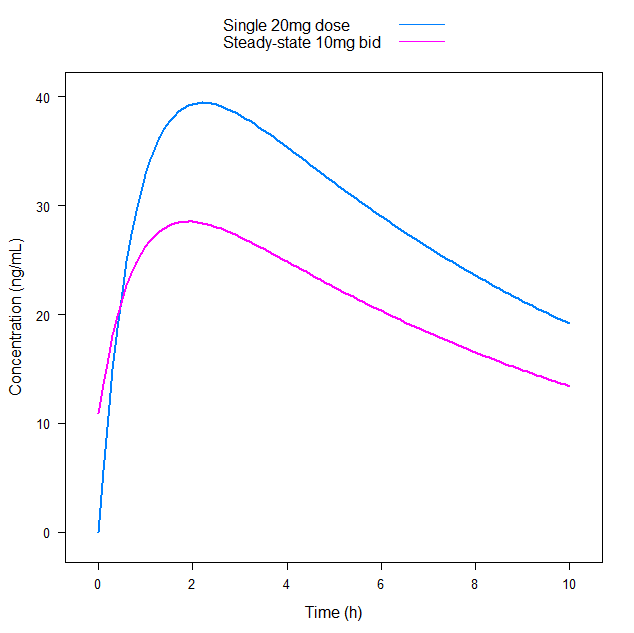


.

1. Performed twice at baseline. [↑](#footnote-ref-1)
2. Training session, except pharmacoEEG. [↑](#footnote-ref-2)
3. Performed twice at baseline. [↑](#footnote-ref-3)
